# Pilot study on the feasibility of shape memory alloy implantation for Vancouver type B1 periprosthetic femoral fractures in a canine model: a step toward advancing treatment modalities

**DOI:** 10.1186/s13018-024-05011-4

**Published:** 2024-08-27

**Authors:** Hyunsoo Kim, Kyu-Won Kang, Timofey Chekalkin, Jang-Woo Park, Hye-Kyung Chung, Byung-Jae Kang, Sung-Woo Choi

**Affiliations:** 1 Department of Veterinary Clinical Sciences, College of Veterinary Medicine and Research Institute for Veterinary Science, Seoul National University, Seoul, 08826 Korea; 2 BK21 FOUR Future Veterinary Medicine Leading Education and Research Center, Seoul National University, Seoul, 08826 Korea; 3 Research and Development Center, TiNiKo Company, Limited, Osong, 28164 Korea; 4 Korea Radioisotope Center for Pharmaceuticals, Korea Institute of Radiological & Medical Sciences, Seoul, 01812 Korea; 5 Department of Orthopedic Surgery, Soonchunhyang University College of Medicine, Seoul, 04401 Korea

**Keywords:** Hip arthroplasty, Vancouver classification type B1, Shape memory alloy, Cerclage cable, Canine

## Abstract

**Background:**

Cerclage wiring is commonly used for treating fractures; however, it has several limitations, including mechanical weakness, decreased blood circulation, and technical complexity. In this study, we developed an implant using a shape memory alloy (SMA) and tested its efficacy in treating Vancouver type B1 (VB1) periprosthetic femoral fractures (PFFs) in a canine model.

**Methods:**

The mid-diaphyseal fracture models underwent reduction via the SMA plate (SMA group) or the cerclage cable plate (cable group) method in randomly selected pelvic limbs. An intraoperative evaluation was conducted to assess the surgical time and difficulty related to implant fitting. Clinical assessments, radiography, microcomputed tomography (micro-CT), histological analysis, positron emission tomography (PET)/CT, and galvanic corrosion analysis were conducted for 52 weeks to evaluate bone healing and blood perfusion.

**Results:**

The results for bone healing and blood perfusion were not significantly different between the groups (*p* > 0.05). In addition, no evidence of galvanic corrosion was present in any of the implants. However, the median surgical time was 75 min (range, 53–82 min) for the SMA group and 126 min (range, 120–171 min) for the cable group, which was a statistically significant difference (*p* = 0.0286).

**Conclusions:**

This study assessed the ability of a newly developed shape memory alloy (SMA) to treat VB1 periprosthetic femoral fractures (PFFs) in canines for over a 52-week period and revealed outcomes comparable to those of traditional methods in terms of bone healing and mechanical stability. Despite the lower surgical complexity and potential time-saving benefits of this treatment, further research is needed to confirm its efficacy.

**Supplementary Information:**

The online version contains supplementary material available at 10.1186/s13018-024-05011-4.

## Introduction

There has been growing demand for improved mobility and quality of life in patients following hip arthroplasty in recent years. A study reported that more than two million people underwent surgery in the United States in 2010 [1]. However, surgical complications, which often result in unfavorable outcomes, are problematic [2, 3]. In particular, the development of intra- or postoperative periprosthetic femoral fractures (PFFs) is a possible problem during primary hip arthroplasty, and identifying PFFs is challenging for surgeons and medical teams [4, 5]. PFFs are classified according to the Vancouver classification [5, 6], and type B1 (VB1) refers to fractures near or just distal to a well-fixed femoral stem [7, 8].

Open reduction and plate fixation have been used for VB1-related PFF repair [9, 10]. However, plate fixation alone results in reduced mechanical strength since standard bicortical screws can be difficult to use because of the intramedullary hip prosthesis in the proximal region of the femur [11, 12]. Therefore, several methods have been used to increase fixation strength, and cerclage cables placed over allograft struts and locking or nonlocking unicortical plates are available options [8]. Recently, a study designed a tangential locking attachment plate for bicortical screws [13]. However, the gold standard method for fixing the proximal region is still elusive, and the common surgical plan involves a combination of cerclage cables and plates [14].

To date, the cerclage wiring method has inherent limitations, including potential blockage of the blood supply due to damage to blood vessels from the elevation of surrounding muscles and the centripetal wrapping of bone [15–18]. To overcome these disadvantages, we designed a nickel-titanium shape memory alloy (NiTi SMA) implant based on its C-shaped characteristics; this material can be widened below an alloy-specific temperature and can grasp the bone as it returns to its original shape at body temperature. [19–21]. Our previous study confirmed that the C-shaped SMA was biomechanical superior to the cerclage cable in VB1 PFF repair using artificial femurs [21].

In this pilot study, we aimed to assess the performance of SMA implants by using a canine model. We hypothesized that the SMA method would have (1) the ability to preserve blood circulation around the osteotomy site due to less damage to soft tissues and (2) sufficient mechanical stability compared to the cable method. In addition, we assessed the biocompatibility of galvanic corrosion between SMA implants and titanium alloy plates.

## Methods

### Animal grouping

The study included four healthy male beagles, all 1 year old, with an average weight of 10.75 (range, 9.7 to 11.5) kg. Each dog was housed individually in a cage and provided with sufficient dry food and water. The dogs underwent surgery on each pelvic limb via different methods and were divided into two groups according to the methods used: the SMA group (SMA-plate method, *n* = 4) and the cable group (cerclage cable-plate method, *n* = 4). At 52 weeks after surgery, all dogs were euthanized with 10% potassium chloride under intravenous (IV) propofol (6 mg/kg) induction and general anesthesia with isoflurane.

### SMA preparation

The raw material used in this study was a medical-grade nickel-titanium shape memory material (nitinol) that met the ASTM F2063-18 standard. The composition ratio of nickel (Ni) to titanium (Ti) in the nitinol alloy was 55:45 (weight%). A 2 mm-thick nitinol plate was obtained and subjected to wire cutting according to the designed procedure. Subsequently, shape memory heat treatment was performed in the temperature range of 300 °C to 600 °C to form the final product. Afterward, pickling treatment was performed. The surface of the nitinol product was pickled using a solution composed of water, nitric acid, and hydrofluoric acid at a ratio of 150:3:1. After pickling, the product was washed under running water for 30 min. Immediately before implantation into animals, the nitinol product was sterilized for 1 h at a temperature of 131 °C using an autoclave.

**Fig. 1:**
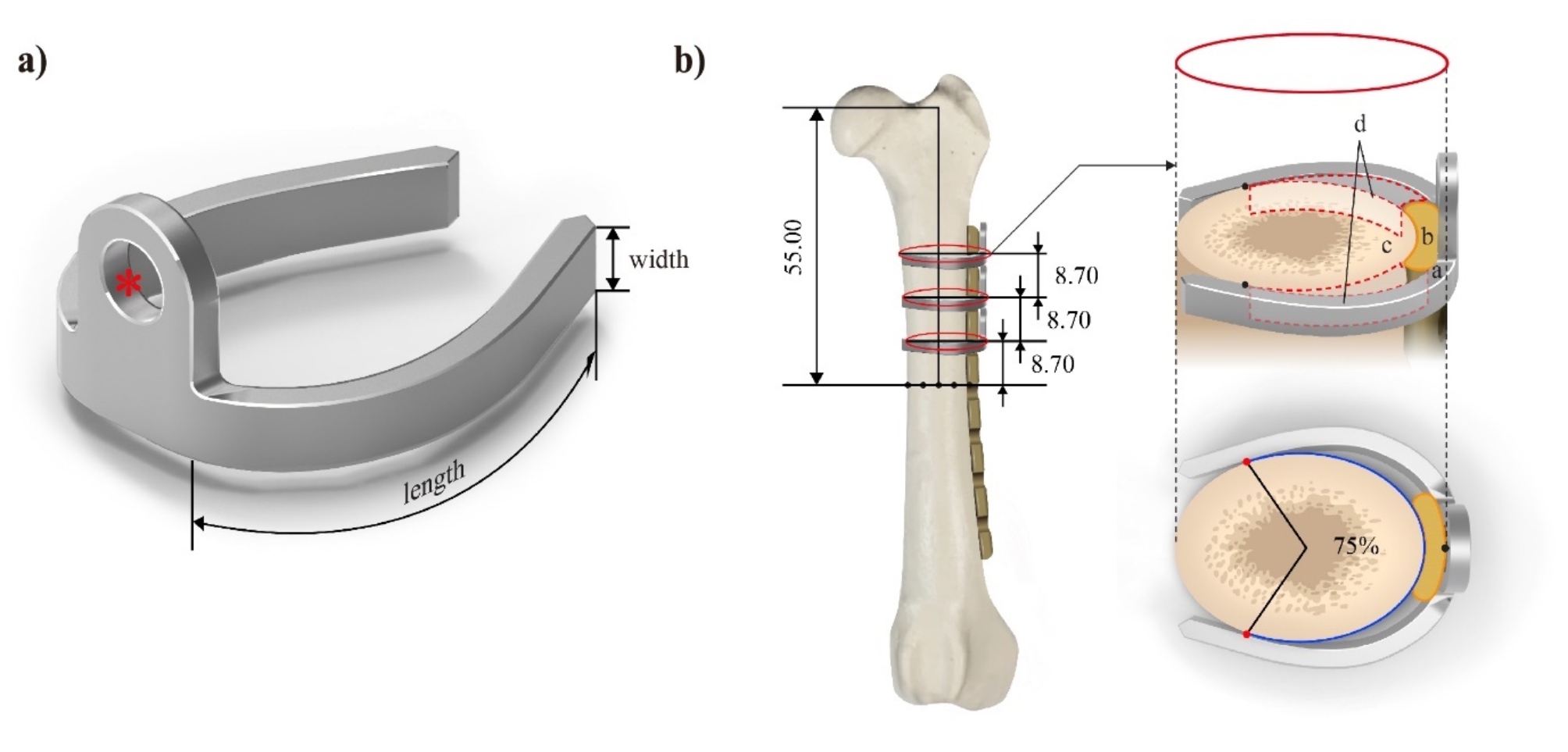
(**a**) Shape memory alloy (SMA) design. The SMA implant was designed on the basis of a previous biomechanical study [22]. The device consisted of a central part with a hole for the cortical screw (red star) and arms that functioned to grasp a bone. The width of the arms was determined to fit the plate notch. (**b**) The arm-bone contact points of an SMA implant. Based on computed tomography (CT), we induced a fracture (dotted line) at 55.00 mm from the craniomedial junction of the greater trochanter, set 8.70 mm as the distance between the center of the screw hole of the plate. The SMA implant made three points of contact with the bone and plate at three levels covering 75% of its circumference. a: SMA, b: plate, c: femoral cortex, d: bone-SMA interspace

Nickel-titanium shape memory alloy (NiTi SMA) implants were designed based on a previous biomechanical study [21]. Preoperative computed tomography (CT) was conducted for each dog to aid in the personalized planning of SMA implants. These implants comprised arms and a screw hole (Fig. 1a).

The arms were subjected to three points of contact upon application, with customized contact points covering 75% of the circumference at each level (Fig. 1b). The implant design included a 2.7 mm cortical screw hole, 3.15 mm arm width to fit the plate notch, and an overall implant thickness of 2 mm.

### Surgical procedure

All surgical procedures were conducted under general anesthesia using isoflurane via the administration of IV meloxicam (0.2 mg/kg) as an analgesic. Additionally, IV cefazolin (30 mg/kg) was administered beginning 1 h before surgery and every 90 min until the end of surgery.

The dogs were placed in lateral recumbency for the operation, and the femoral shaft was approached. First, an incision was made along the craniolateral border of the femoral shaft from the greater trochanter to the patella. Then, an incision was made between the fascia lata and the biceps femoris. The muscles were retracted, the fascia lata and vastus lateralis were retracted cranially, and the biceps femoris was pulled back caudally to expose the femoral shaft. A transverse osteotomy was created using an electrical saw (Colibri II, Synthes GmbH, Oberndorf, Switzerland) for the VB1 fracture model [6]. The osteotomy sites were created at an average distance of 55 mm from the craniomedial junction of the greater trochanter based on preoperative CT. Next, the fragmented bones were aligned using Kern bone-holding forceps. The four dogs underwent surgery on each pelvic limb using different methods. One limb was treated with the SMA-plate method, and the other was treated with the cable-plate method.

In the cable group, the proximal portion of the femur was stripped circumferentially. Then, a precontoured plate based on 3-dimensional (3D)–printed bones was applied along the lateral side of the shaft, and three cortical screws were inserted bicortically on the distal side. Finally, three single-loop cerclage cables (Ø 1.0 mm, SUS316L, DePuy Synthes Co., Paoli, PA, USA) were placed beyond plate notches with 4-Nm force tension for reinforcing the proximal part of the plate applied serially to the proximal first, second, and third notches. Three unicortical screws were inserted on the proximal side under the same conditions for both methods.

In the SMA group, three bicortical screws were first inserted into the distal segment, and the proximal segment was elevated only enough to allow the SMA arms to be inserted. The SMA, which was previously placed in an implant-cooling former at 4 °C for transformation, was widened using a needle holder and applied to the proximal first notch of the plate. Similarly, second and third proximal SMAs were applied. The osteotomy site and SMAs were confirmed to be stable, and 2.7 mm cortical screws were inserted unicortically into each hole.

The surgical site was closed using standard techniques. A continuous suture with PDS 3 − 0 (Ethicon, Johnson & Johnson, Somerville, NJ, USA) was applied to the fascia along the cranial border of the biceps femoris muscle. This was followed by continuous suturing of the fascia and subcutaneous fat along the incision line. The skin was closed with nylon sutures. The skin incision line was covered by a Mepilex Border (Mölnlycke Health Care, Gothenburg, Sweden) and dressed daily for one week.

Postoperative radiographs of eight limbs were obtained, and the dogs were placed in individual cages for recovery. For postoperative use, oral cefadroxil (30 mg/kg) and oral famotidine (0.5 mg/kg) were administered every 12 h for 5 days, and meloxicam (0.1 mg/kg) was subcutaneously injected once a day for analgesia every 24 h for 5 days.

### Intraoperative evaluation

#### Difficulty rating

The difficulty of each case was rated by a single orthopedic clinician based on the degree of soft tissue stripping, procedural complexity, and implant fitting after implantation. The degree of soft tissue stripping referred to the extent of manipulation or dissection required during the procedure, with greater stripping considered more difficult. Procedural complexity encompassed the overall intricacy of the surgical steps and the challenges encountered during the operation. Implant fitting after implantation was evaluated based on how well the implant fit post-implantation, considering the adjustments needed for optimal placement. To quantify these assessments, a three-point scale was used: 1 (simple), 2 (moderate), and 3 (complicated) (Supplementary Table S1).

#### Surgical time

The surgical time was measured from anesthetic induction to skin closure using a stopwatch with an accuracy of 0.01 s.

### Clinical and radiographic evaluations

All dogs underwent clinical and radiographic evaluation by the same trained orthopedic clinician preoperatively and at 4, 8, 16, 24, and 52 weeks postoperatively. Weight-bearing ability was assessed using a five-point scale: (1) full weight-bearing for > 30 s, (2) full weight-bearing with resistance for > 30 s, (3) weight-bearing for < 30 s, (4) weight-bearing for < 10 s, and (5) refusal to raise the contralateral limb. Fracture site tenderness was evaluated using a five-point scale: (1) no pain, (2) head turn, (3) limb withdrawal, (4) vocalization, and (5) refusal to allow palpation [23]. The total sum of the two criteria was used for the statistical analysis. Gait analysis was performed using a pressure walkway system (Strideway, Tekscan, Boston, MA, USA) to measure the weight distribution. The peak vertical force (PVF) was recorded for each limb, and the weight distribution (WD) was calculated as follows: (PVF of the affected limb/PVF of all four limbs) × 100.

Orthogonal X-ray images were obtained to assess alignment, implant failure, fracture, and bone healing at each follow-up.

### Sample preparation and histologic observation

Four dogs were euthanized with 10% potassium chloride IV at 52 weeks postoperatively, following induction with propofol and anesthesia with isoflurane. Bilateral femurs were harvested after sacrifice. The femoral shaft was accessed via a lateral approach with the dog in lateral recumbency. Discoloration and inflammation in the peri-implant tissue were initially evaluated, along with implant loosening and impingement. Bone samples were carefully exposed from the surrounding soft tissue by periosteal stripping and then harvested using an electrical saw at the level of the lesser trochanter. Implants were disassembled, and the remodeled bone and fibrous tissue overlying the implants were carefully removed. The samples were fixed in buffered formalin (10%, pH 7.4). After rinsing the debris with 70% alcohol and gauze, the implants were stored. Following micro-CT analysis, specimens were stained with hematoxylin and eosin (HE) and Masson’s trichrome (MT) and then decalcified. Longitudinal sections were analyzed to evaluate the bone healing process within the fracture gap.

### Micro-CT evaluation

Micro-CT (SkyScan, Bruker, Belgium) was used to evaluate bone healing precisely at the fracture site. A cylindrical volume of interest (VOI) was defined as extending 5 mm proximal and distal to the fracture line up to 10 mm. 3D reconstructions were performed using the SkyScan NRecon program. The degree of callus bridge formation and mineralization was assessed using 3D reconstruction for bone remodeling analysis. Morphometric parameters, including bone mineral density (BMD), bone volume/total volume (BV/TV), and polar moment of inertia (PMI), were measured using SkyScan Dataviewer and SkyScan Ctan software (Bruker, Belgium) to evaluate late-stage parameters [24–27].

### Positron emission tomography/computed tomography (PET/CT)

A clinical PET/CT system (Discovery STE, GE Healthcare, Milwaukee, WI, USA) was used for dynamic PET imaging. The influx rate (Ki) of [18 F]NaF was estimated as an index of functional recovery of bone. In a previous study [28], the Ki value of [18 F]NaF obtained from kinetic analysis via PET imaging could provide as much information as blood flow in the evaluation of bone disorders. Furthermore, one study has estimated the blood flow using dynamic images of first-pass [18 F]FDG [29]. The kinetic characteristics of FDG and NaF are similar. In this study, the image acquisition duration of the first 3 min after injection was chosen for further kinetic analysis of the estimated K1 value.

On the fourth to fifth days after the operation, PET/CT imaging was performed at the Korea Radioisotope Center for Pharmaceuticals. All the procedures were conducted under general anesthesia using isoflurane with the induction of IV alfaxalone (2 mg/kg). CT images were acquired at an X-ray voltage of 120 kVp and an anode current of 70 mA. The CT images were used for PET image reconstruction, including corrections for attenuation and scatter. The dynamic PET images were acquired for approximately 1 h. The PET scan started 10 s before the injection of 185 MBq of [18 F]NaF. [18 F]NaF was injected into the cephalic vein via an intravenous catheter for 30 s using an injector. Longitudinal and transaxial views were obtained at the level of the proximal cable or SMA implant application and at the level of the fracture line. Kinetic analysis was applied to estimate the K1 value around the femur, where the cerclage cable wiring and SMA were located, using a dynamic image of [18 F]NaF. The input function was obtained by drawing a circular region of interest (ROI) with a radius of 3 mm on the abdominal aortic region of the PET images. Additional circular ROIs with different radii were placed in the regions at the implant application level (*r* = 12 mm) and fracture line level (radius = 10 mm). To estimate the K1 value, nonlinear fitting based on a 1-tissue compartmental model was used.

### Scanning electron microscopy/energy dispersive spectroscopy (SEM/EDS)

The plate surface and implant interface were examined for galvanic corrosion using scanning electron microscopy (SEM; CUBE-2, EMCRAFTS, Hanam, South Korea) and energy-dispersive spectroscopy (EDS; X Flash Detector 630 H, Bruker, M, USA). Galvanic corrosion is defined as the electrochemical degradation derived from the joining of two or more different or dissimilar alloys [19, 30]. We visually observed galvanic corrosion around the implant by separating the skin, subcutaneous tissue, and muscles layer by layer and looking for discoloration, granulation tissue, and necrosis. All SEM images were obtained using secondary electrons emitted from the sample after collision with a primary electron. The EDS quantitative analysis yielded ZAF-corrected elemental information through energy dispersion X-ray spectroscopy and prevented the absorption effect due to close contact with the sample. Galvanic corrosion was comprehensively confirmed based on the results from the above tests.

### Statistical analysis

Statistical analysis was performed using GraphPad Prism V 7.00 (GraphPad Software, Inc., USA).

#### General and clinical assessment

The surgical time and difficulty level are expressed as medians (ranges). The Mann‒Whitney U test was used to assess the statistical significance of differences between groups. A value of *p* < 0.05 indicated statistical significance.

#### Micro-CT and PET/CT evaluation

The results are presented as the mean ± standard deviation (SD). The Mann‒Whitney U test was used to assess the statistical significance of differences between groups. Differences were considered to be statistically significant at *p* < 0.05.

#### Galvanic corrosion evaluation

The quantitative EDS results are expressed as the mean ± standard deviation (SD). Two-way ANOVA was performed to assess statistical significance. Differences were considered to be statistically significant at *p* < 0.05.

## Results

### General observations

The mean surgical time was 126 min (range, 120–171 min) in the cable group and 75 min (range, 53–82 min) in the SMA group. The median (range) difficulty score was 2 (range, 1–3) in the cable group and 1.5 (range, 1–2) in the SMA group. All the implants were fitted without instability during surgery. The only significant difference between the groups was observed in terms of surgical time (*p* = 0.0286).

No major complications, such as surgical site infection or implant failure, required revision surgery in either group. A single case of cable loosening was identified in the cable group, and a unicortical screw was used to fix the most distal screw hole in the distal region in one case in the SMA group.

### Clinical assessment

Upon subjective clinical evaluation, the cable group exhibited a median total score of 3 (range, 2–3.5) at 2 weeks, 2.5 (range, 2–3) at 8 weeks, and 2 (all) at subsequent serial follow-ups. Similarly, in the SMA group, the median total score was 3 (range, 2–3.5) at 4 weeks, 2.25 (range, 2–2.5) at 8 weeks, and 2 (all) at subsequent serial follow-ups. No significant differences were observed between the two groups (*p* > 0.05).

In terms of gait analysis, the cable group exhibited a mean WD of 16.00 ± 2.94% at 4 weeks, 20.50 ± 1.29% at 16 weeks, 19.25 ± 1.26% at 24 weeks, and 19.75 ± 2.22% at 52 weeks. Moreover, the SMA group displayed a mean WD of 13.75 ± 3.20% at 4 weeks, 19.50 ± 1.92% at 16 weeks, 19.00 ± 0.81% at 24 weeks, and 19.75 ± 1.90% at 52 weeks. No significant differences were observed between the two groups (*p* > 0.05).

**Fig. 2:**
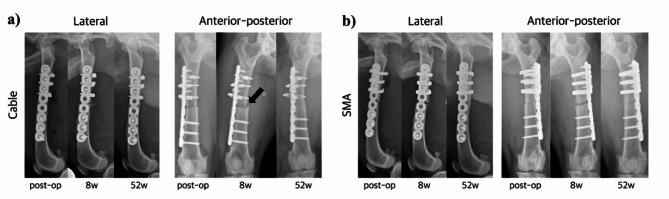
Radiographic observations. We compared the cerclage cable and shape memory alloy (SMA) using X-ray images. (**a**) Cable, (**b**) SMA. At 8 weeks, both groups exhibited callus formation without cortical connectivity. At 52 weeks, bone union was recognized with cortical connectivity. The black arrow indicates misalignment due to cable loosening. Left: postoperative, middle: 8 weeks, right: 52 weeks

Radiographic imaging revealed callus formation in three out of four cases in the cable group at 4 weeks. At 8 weeks, one out of four cases had cortical connectivity, which progressed to bone union at 16 weeks. At 24 weeks, one case had more bone unions, and the remaining two out of four limbs had sclerotic changes at the ends of the fragments and progressive hypertrophic nonunion. At 52 weeks, bone union was identified in two out of the four cases in the cable group, including two nonunion cases (Fig. 2a).

In the SMA group, progression parallel to that in the cable group was observed until 24 weeks. Bone union was more commonly achieved in one limb at 42 weeks. Eventually, at 52 weeks, bone union was identified in three out of four cases in the SMA group, including one case with delayed union and one with nonunion (Fig. 2b).

In summary, the dogs in both groups demonstrated partial weight-bearing in their bilateral pelvic limbs for approximately 6–12 weeks, and all the dogs eventually exhibited normal weight-bearing ability after 16 weeks with gradual cortical connectivity. Furthermore, gait analysis revealed a normal WD after 16 weeks, which was consistent with the subjective evaluations.

### Bone healing evaluation using micro-CT and histological analysis

During harvest, no discoloration or inflammation was observed in the peri-implant tissue. All plates were encapsulated with soft or hard callus tissues, which were carefully separated to obtain the underlying bone samples.

**Fig. 3:**
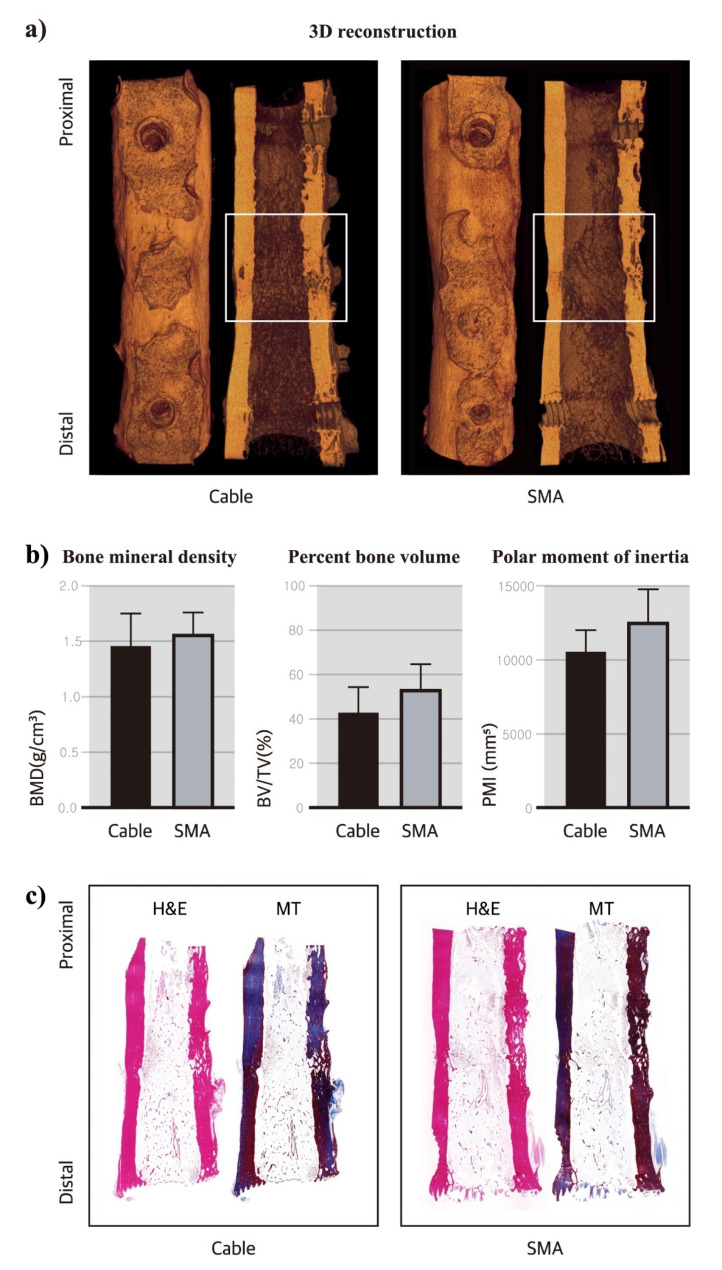
Microcomputed tomography (CT) and histological evaluation. (**a**) Longitudinal images and 3D reconstruction of the femur using micro-CT. (**b**) Comparison of mean bone mineral density (BMD), percent bone volume (BV/TV), and polar moment of inertia (PMI) between the cable and shape memory alloy (SMA) groups. The SMA group exhibited higher values than the cable group, but the difference was not statistically significant (*p* > 0.05). (**c**) Histological morphology with hematoxylin and eosin (HE) and Masson’s trichrome (MT) staining, showing bone remodeling in the osteotomy region with cortical longitudinal continuity. White square indicates cylindrical volume of interest (VOI) set at 10 mm intervals

Micro-CT analysis was conducted to evaluate the bone healing process in the two groups of femurs subjected to different treatment methods. In the cable group, two femurs exhibited cortical bone union; however, the cortex on the plate side was coarse (Fig. 3a, left).

Similarly, in the SMA group, the density of the two femurs with bone union decreased on the plate side (Fig. 3a, right). In cases of delayed union, a thin osteotomy line with lamellar bones was observed in the far cortex of the plate. In both groups, femoral nonunion exhibited signs of bone healing, with the fracture gap showing the sclerotic ends of the bone fragments.

**Fig. 4:**
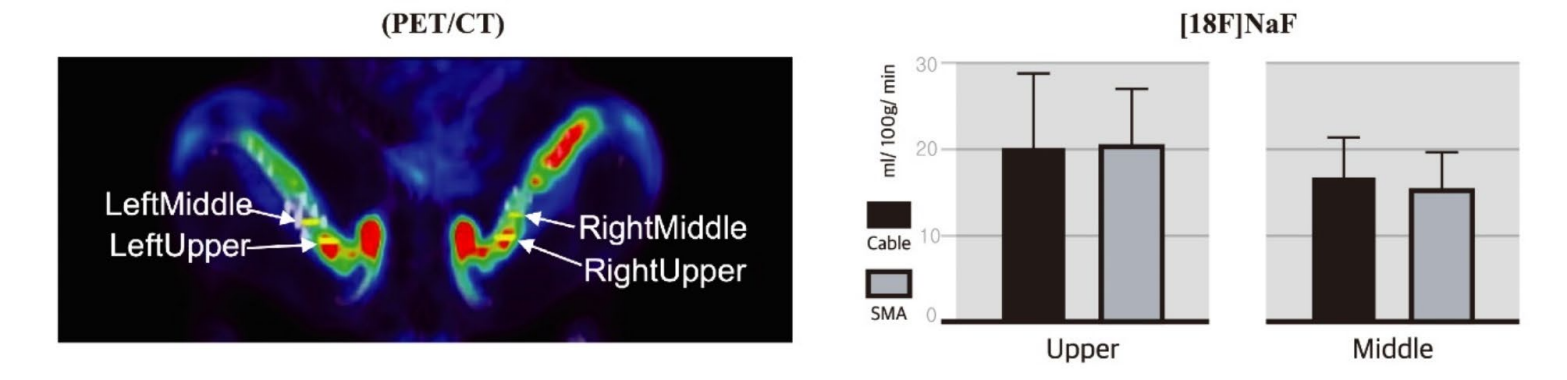
Positron emission tomography/computed tomography (PET/CT) for bone blood supply. Evaluation of the degree of blood circulation in the middle and upper parts of the bone between the cable and shape memory alloy (SMA) groups. There were no statistically significant differences between the groups (*p* > 0.05)

A quantitative evaluation of the BMD, BV/TV, and PMI was also conducted to compare the two groups. The cable group had a mean BMD of 1.46 ± 0.29 g/cm^3^, a mean BV/TV of 42.51 ± 11.60%, and a mean PMI of 1050.66 ± 1421.25 mm^5^. The corresponding values for the SMA group were 1.56 ± 0.20 g/cm^3^, 53.00 ± 11.46%, and 28 ± 2323.28 mm^5^, respectively (Fig. 4b). None of the values differed significantly between the groups (*p* > 0.05).

Histological evaluation was conducted following the micro-CT analysis. In the cable group, bone remodeling was observed, with a well-organized cortex and bone marrow cavity in two out of four cases. In the remaining bones, the cortex was coarse, and a fibrotic soft tissue bridge was present between the osteotomy gaps. Similarly, in the SMA group, bone remodeling was noted; however, in cases of delayed union, the thin osteotomy gap consisted of fibrotic and non-mineralized components.

### PET/CT

PET/CT analysis revealed that the mean K1 value of [^18^F]NaF was 20.03 ± 8.74 mL/100 g/min in the proximal femoral region and 16.65 ± 4.68 mL/100 g/min in the middle femoral area in the cable group. In the SMA group, the mean metabolic rate was 20.38 ± 6.59 mL/100 g/min in the proximal femoral region and 15.30 ± 4.30 mL/100 g/min in the middle femoral area. There were no significant differences in blood flow between the two groups (*p* > 0.05) (Fig. 4).

### Galvanic corrosion evaluation

**Fig. 5:**
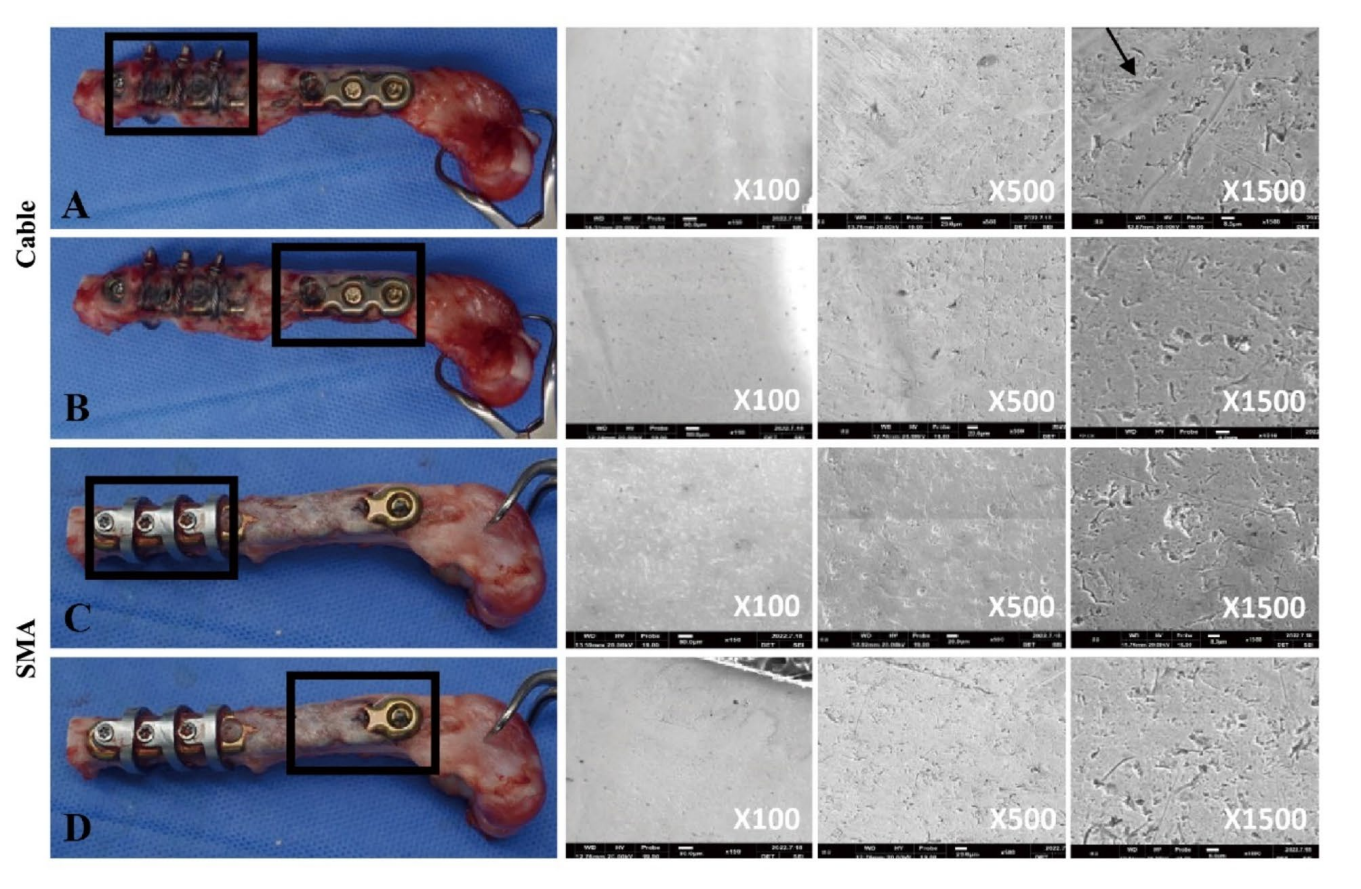
Evaluation of galvanic corrosion. We assessed galvanic corrosion using visual inspection and scanning electron microscopy (SEM). We found no traces of galvanic corrosion on titanium alloy plates in contact with the cable and shape memory alloy (SMA) implant. **A**: titanium alloy plate in contact with the cable, **B**: titanium alloy plate not in contact with the cable, **C**: titanium alloy plate in contact with the SMA implant. **D**: titanium alloy plate surface not in contact with the SMA implant. Black arrow: trace of peeling; black square: plate region for SEM analysis

SEM/EDS was performed on all devices after disassembly from the bone. The cable group exhibited surface damage at the cable-plate interface, such as cracking and peeling, visible at 1500X magnification (Fig. 5).

However, the EDS analysis revealed no differences in the elemental composition between the interface and the noncontact region. In contrast, the SMA group showed no surface damage or elemental changes at the SMA-plate interface or noncontact plate area (Fig. 5 and Supplementary Table S2). These findings were consistent with the gross observations at retrieval.

## Discussion

In this study, we compared a newly devised SMA method with a protocol involving cerclage cable wiring placement over a plate, to verify the clinical usability of the SMA in an in vivo environment. Our results showed a significant difference in surgical time between the groups, whereas soft tissue damage and mechanical strength did not differ. The SMA group demonstrated satisfactory functional recovery without major complications over a 52-week follow-up. These findings suggest that the SMA method may be a viable alternative to cerclage cable wiring with the potential benefit of procedural convenience.

In this study, we designed an SMA implant that could be inserted using a cortical screw. Previous research by Zhao et al. [31] used an SMA embracing a fixator with spiked arms as a substitute for the cable-plate system in PFFs. However, we considered that the large size of the implant would be disadvantageous. Therefore, we selected individual SMA clip types for versatility. In our preliminary study, we conducted an experiment on two dogs using one screw-inserting SMA and two C-shaped SMAs applied to the proximal femoral region over the plate. However, one dog experienced SMA positional failure 7 days after surgery (Supplementary Fig. S1), and all the fracture gaps progressed to hypertrophic nonunion within 5 months. We concluded that the preversion SMA had insufficient fixation strength, and we revised the design as follows. First, we lengthened the SMA arms to cover 75% of the femoral circumference, up from 60%, to provide more stable bone contact. Second, we increased the number of screw-inserting SMAs to three because we believed that the cortical screw would prevent displacement in the axial direction of the SMA implant [32].

Intraoperative evaluation revealed that the time required for the procedure was significantly shorter in the SMA group than in the control group and that the SMA procedure was simple to apply. Cable wiring typically involves seven steps: (1) placing the wire passer around the bone; (2) inserting the cable into the wire passer; (3) removing the wire passer; (4) inserting the crimp; (5) tightening with a tightener; (6) fastening with a crimp; and (7) cutting the wire. In contrast, SMA implantation involves three steps: (1) precooling the implant in cold water before surgery, (2) implanting it over a plate, and (3) irrigating the SMA with warm fluid. The placement time and process for the SMA were short and straightforward. However, handling the SMA for a few minutes could have caused the implant to shrink before application, potentially adding additional time to the implantation process until the users became accustomed to the new implant.

In this study, radiographs confirmed nonunion of the three limbs in both groups. We believe that this may have been due to the inability of the plate to sufficiently compress the fragments, resulting in a gap with high strain [33, 34]. There is also the possibility of thermal osteonecrosis at the fragment ends caused by iatrogenic orthopedic sawing during fracture modeling [35]. To date, a reliable biomechanical assessment of stiffness at the fracture site has not been established as a clinical tool. Therefore, evaluations of bone healing in a clinical setting are still considered useful, not only through radiographic data but also through assessments of weight-bearing status and pain upon palpation in physical examinations [36]. According to our clinical evaluation, no significant differences were found between the groups, and both groups exhibited functional recovery after 16 weeks, with normal levels of tenderness, weight-bearing, and gait analysis.

We used both micro-CT and radiography to evaluate bone healing. Micro-CT is a standard tool for measuring and visualizing bone; however, there is no consensus on the optimal micro-CT-derived parameters for evaluating bone quality. We considered BMD to be the main factor [37, 38] and selected BV/TV and the PMI as late-stage parameters based on several studies [24, 25]. BV/TV was chosen as a late-stage parameter because our evaluation occurred at 52 weeks. Therefore, since the callus at the initial fracture site is usually absorbed and replaced by lamellar bone within 4 months [36], we believed that the BV/TV, as a ratio of bone volume, was a suitable measure of fusion quality. The PMI, which measures resistance to bending stress from multiple angles, was also chosen as a late-stage parameter because it takes at least 1 year for bone quality to return to normal [39, 40].

We aimed to assess the degree of soft tissue damage, including blood blockage, as these are important biological factors in bone healing after fractures [36, 41]. For this purpose, we chose PET/CT, which is noninvasive and provides anatomical information [42–46]. Previous studies have shown that bone healing can be affected by the invasiveness of the fixation method used [40, 42, 47–49]; however, our PET/CT results showed no significant differences between the groups. Nevertheless, we believed that the SMA method could have two advantages. First, while cerclage cable wiring tightens the bone and plate, causing all parts to be in contact, the SMA method has points of contact that create space between the bone and the SMA interface (Fig. 1b), allowing preservation of the periosteum and surrounding soft tissue. Second, the application of SMAs does not require extensive circumferential soft tissue elevation. Cerclage cable wiring requires a path inside muscles inserted into the pectineal line or linea aspera, which are the routes of the main nutrient artery for the femur [50]. Based on the above considerations, the SMA method may be advantageous for bone healing because of its superior blood circulation potential.

SMAs exhibit excellent biocompatibility due to their high corrosion resistance [19, 51]. However, the concurrent use of different metals sometimes occurs in orthopedic cases, as in this study. Galvanic corrosion can cause side effects such as inflammation, interference with osseointegration, and granulation formation [19, 51]. We investigated the difference in galvanic corrosion between the SMA and titanium alloy plates and found no evidence of galvanic corrosion for up to 52 weeks. Surface irregularities resulting from cable tightening may disrupt oxide film formation and promote corrosion [52–54]. In fact, the cable implantation caused plate fretting when the plate was tightened with a multifilament wire; however, no scratching was observed in the SMA group. Since SMAs tighten naturally with temperature, the metal surface oxide layer is less likely to collapse, with fewer contact points on the SMA arms.

One of several limitations of this study was the lack of a control group. Ideally, groups would have been compared separately by the method used; however, grouping with an increased number of dogs was challenging due to the evolving interest in animal ethics [55]. Therefore, we compared groups by applying each method to the same dog. Although obtaining statistically significant differences between the groups was difficult, this experimental model and analytical approach will serve as a foundation for future research.

The second limitation of this study is the absence of biomechanical testing specifically for the SMA implants with screw insertion used in this experiment. However, it is important to note that the current SMA design is an evolved form of the previously tested C-shaped SMA. By incorporating an additional screw insertion hole, the design aims to enhance the bonding strength between the plate and the SMA, thereby reducing the instability between the implants. While we believe this represents an advancement, we acknowledge the importance of evaluating the rotational stability introduced by the screw insertion. Future research will consider conducting biomechanical tests to assess this aspect more thoroughly.

Given that PFF treatment can lead to catastrophic complications [2, 3, 56], additional studies focusing on femoral stem insertion for VB1 PFF, along with in vivo preclinical studies, are necessary before human clinical trials. While the results for the SMA group were encouraging, the sample size was relatively small. Therefore, further long-term, controlled evaluations of these implants are essential.

## Conclusion

This pilot study assessed the clinical usability of a newly manufactured SMA in an in vivo environment for the surgical treatment of VB1 PFF over a 52-week period. Compared to the cable group, the SMA group had a significantly shorter procedure time due to the simplicity of the protocol. While the SMA method showed potential as a viable option for VB1 PFFs, a larger-scale study is warranted in the future.

### Electronic supplementary material

Below is the link to the electronic supplementary material.

Supplementary Material 1

Supplementary Material 2

Supplementary Material 3

## Data Availability

No datasets were generated or analysed during the current study.
