# Supplementary material for: Pilot study on the feasibility of shape memory alloy implantation for Vancouver type B1 periprosthetic femoral fractures in a canine model: a step toward advancing treatment modalities
Source: J Orthop Surg Res. 2024 Aug 27;19:510. doi: 10.1186/s13018-024-05011-4 (PMC11348569; doi:10.1186/s13018-024-05011-4)
Supplement: Supplementary file 1 — Supplementary Material 1 [file 13018_2024_5011_MOESM1_ESM.docx]

**Supplementary Figure S1**: Revision of the shape memory alloy (SMA) design. (a) Two C-shaped SMAs and screw screw-inserting SMA application and failure (left and middle). (b) Revised SMA application (right). Note: The revised design lengthened and narrowed the SMA arms to improve fixation.
