# Supplementary figures and images for: Pilot study on the feasibility of shape memory alloy implantation for Vancouver type B1 periprosthetic femoral fractures in a canine model: a step toward advancing treatment modalities
Source: J Orthop Surg Res. 2024 Aug 27;19:510. doi: 10.1186/s13018-024-05011-4 (PMC11348569; doi:10.1186/s13018-024-05011-4)

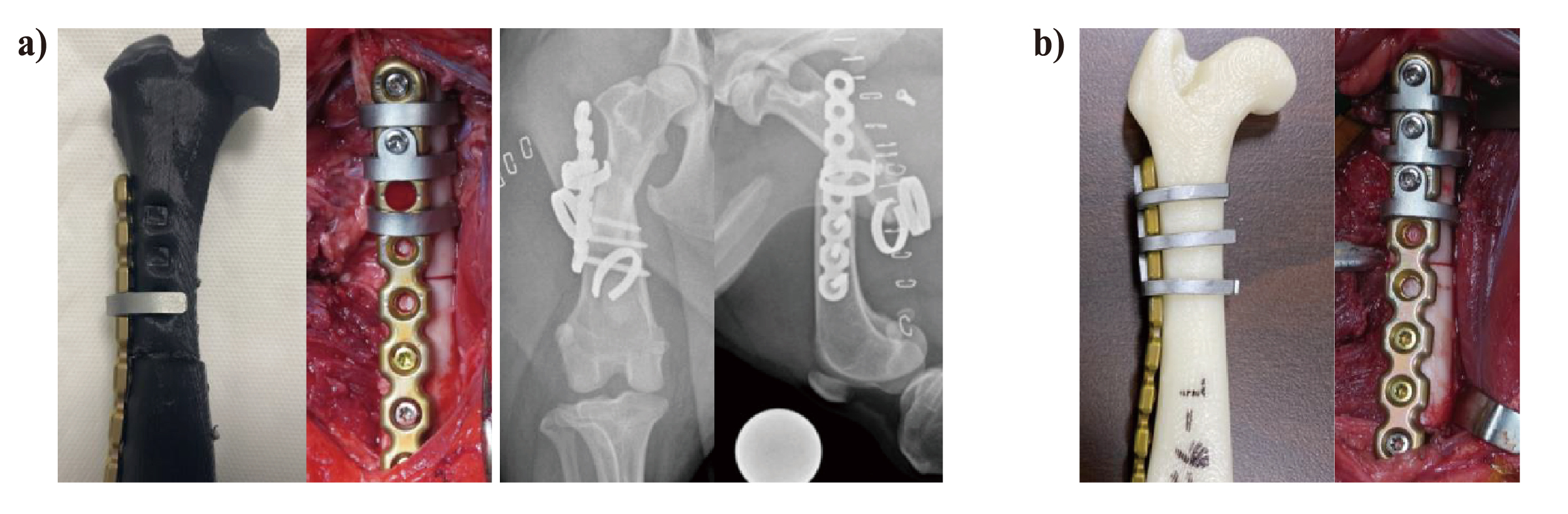

Supplement: Supplementary file 2 — Supplementary Material 2 [file 13018_2024_5011_MOESM2_ESM.jpg]
