# Supplementary material for: Pilot study on the feasibility of shape memory alloy implantation for Vancouver type B1 periprosthetic femoral fractures in a canine model: a step toward advancing treatment modalities
Source: J Orthop Surg Res. 2024 Aug 27;19:510. doi: 10.1186/s13018-024-05011-4 (PMC11348569; doi:10.1186/s13018-024-05011-4)
Supplement: Supplementary file 3 — Supplementary Material 3 [file 13018_2024_5011_MOESM3_ESM.docx]

**Table legends**

**Supplementary Table S1**: Difficulty rating criteria: A three-point scale assessing soft tissue stripping, procedural complexity, and implant fitting

| Rating | Description |
| --- | --- |
| 1 (Simple) | Minimal soft tissue stripping, low procedural complexity, and straightforward implant fitting |
| 2 (Moderate) | Moderate soft tissue stripping, moderate complexity, and some adjustments needed in implant fitting |
| 3 (Complicated) | Extensive soft tissue stripping, high procedural complexity, and significant difficulties in achieving proper implant fitting |

**Supplementary Table S2**: Energy dispersive spectroscopy (EDS) quantitative analysis of interconnection surfaces. We performed EDS to quantify the elements on the connection surfaces. There was no significant difference between implant-plate contact and implant-plate no contact region in the cable and shape memory alloy (SMA) groups (p > 0.05). Element ratio (%), mean ± SD of four plate samples.

| Element | Cable- plate contact | Cable- plate  No contact | SMA-plate contact | SMA-plate  No contact |
| --- | --- | --- | --- | --- |
| C | 2.94 ± 0.99 | 3.45 ± 1.66 | 3.46 ± 1.63 | 4.50 ± 1.39 |
| O | 24.72 ± 1.14 | 24.35 ± 1.60 | 23.81 ± 1.30 | 24.81 ± 0.80 |
| Na | 0.19 ± 0.24 | 0.14 ± 0.19 | 0.12 ± 0.16 | 0.38 ± 0.11 |
| Mg | 0.04 ± 0.06 | 0.03 ± 0.07 | 0.02 ± 0.02 | - |
| Al | 4.01 ± 0.51 | 3.86 ± 0.69 | 3.64 ± 0.26 | 3.68 ± 0.15 |
| Si | 0.04 ± 0.05 | 0.02 ± 0.02 | 0.01 ± 0.01 | 0.02 ± 0.02 |
| P | 0.10 ± 0.04 | 0.11 ± 0.01 | 0.12 ± 0.03 | 0.11 ± 0.03 |
| K | - | 0.01 ± 0.01 | 0.01 ± 0.01 | 0.02 ± 0.01 |
| Ca | 0.01 ± 0.01 | 0.01 ± 0.02 | - | 0.03 ± 0.02 |
| Ti | 65.82 ± 1.22 | 65.86 ± 1.71 | 67.49 ± 1.83 | 64.35 ± 0.91 |
| V | 1.85 ± 0.03 | 1.92 ± 0.33 | 1.84 ± 0.17 | 1.80 ± 0.06 |
| Cr | 0.05 ± 0.06 | 0.06 ± 0.02 | 0.08 ± 0.03 | 0.06 ± 0.05 |
| Mn | 0.03 ± 0.03 | 0.01 ± 0.02 | 0.02 ± 0.03 | - |
| Fe | 0.14 ± 0.07 | 0.11 ± 0.05 | 0.10 ± 0.04 | 0.12 ± 0.02 |
| Ni | 0.02 ± 0.03 | 0.04 ± 0.02 | 0.02 ± 0.02 | 0.02 ± 0.02 |
| Mo | 0.11 ± 0.09 | 0.02 ± 0.02 | 0.01 ± 0.01 | 0.04 ± 0.04 |
| Dy | 0.01 ± 0.02 | - | 0.03 ± 0.04 | 0.01 ± 0.01 |
